# Autosomal dominant intellectual disability

**DOI:** 10.1007/s11825-018-0206-2

**Published:** 2018-10-11

**Authors:** Dagmar Wieczorek

**Affiliations:** Institut für Humangenetik, Universitätsklinikum Düsseldorf, Heinrich-Heine-Universität, Universitätsstr. 1, 40225 Düsseldorf, Germany

**Keywords:** Intellectual disability, Autosomal dominant, *De novo*, Next generation sequencing, Mosaicism, Intelligenzminderung, autosomal-dominant, Geistige Behinderung, *De novo*, Hochdurchsatz-Sequenzierung, Mosaik

## Abstract

Intellectual disability (ID) is a heterogeneous entity defined as a substantial impairment of cognitive and adaptive function with an onset in early childhood and an IQ measure of less than 70. During the last few years, the next generation technologies, namely whole exome (WES) and whole genome sequencing (WGS), have given rise to the identification of many new genes for autosomal dominant (ADID), autosomal recessive (ARID) and X‑linked forms of ID (XLID). The prevalence of ID is 1.5–2% for milder forms (IQ < 70) and 0.3–0.5% for more severe forms of ID (IQ < 50). Up to now, about 650 genes for ADID have been reported and it is expected that there are at least 350 genes still unidentified. Although the ADID genes can easily be classified according to the associated clinical findings, e. g. different kind of seizures, abnormal body measurements, an advanced selection of reasonable genes for analyses is challenging. Many different panels for ID genes have been developed for a first diagnostic step, but more meaningful is the use of trio exome sequencing in individuals with sporadic ID. Using trio WES the mutation detection rate for *de novo* mutations in ID varies from 20 to 60%.

Further research is needed for the identification of the remaining ID genes, a deeply understanding of the pathways and the development of therapies for the mostly rare causes of ID.

## Zusammenfassung

Die Intelligenzminderung, geistige Behinderung, ist eine heterogene Entität, die definiert ist als Beeinträchtigung der kognitiven und adaptiven Funktionen mit einem Beginn im frühen Kindesalter und einem IQ von weniger als 70. Während der letzten Jahre haben die neuen Hochdurchsatz-Sequenzierungsverfahren, die Exom-Sequenzierung und die Genom-Sequenzierung, zur Identifizierung von zahlreichen neuen Genen für die autosomal-dominante, autosomal-rezessive und X‑gebundene Intelligenzminderung geführt. Die Prävalenz für die Intelligenzminderung ist mit 1,5–2 % für milde Formen (IQ < 70) und mit 0,3–0,5 % für schwerere Formen der Intelligenzminderung (IQ < 50) anzugeben. Bis zum jetzigen Zeitpunkt wurden ca. 650 Gene für eine autosomal-dominante Intelligenzminderung publiziert und es wird geschätzt, dass es mindestens noch 350 weitere Gene gibt, die bisher noch nicht identifiziert wurden. Grundsätzlich kann man die Gene nach den assoziierten klinischen Zeichen, z. B. verschiedene Formen der Epilepsie oder auffällige Körpermaße, in Untergruppen einteilen. Trotzdem ist eine sinnvolle Vorauswahl von Genen für die Analyse bei Intelligenzminderung eine große Herausforderung. Es wurden zahlreiche verschiedene Panels für den ersten diagnostischen Schritt entwickelt, dennoch muss man sagen, dass die Trio-Exom-Sequenzierung bei sporadischer Intelligenzminderung die Methode der Wahl ist. Mit dieser Methode kann man eine *de novo* Mutationsdetektionsrate von 20 bis 60 % erreichen.

Weitere Forschungsprojekte sind notwendig und sinnvoll, um die noch nicht identifizierten Gene für die Intelligenzminderung zu finden, die zugrunde liegenden Signalwege zu verstehen und Therapien für die meistens doch sehr seltenen Ursachen der Intelligenzminderung/geistigen Behinderung entwickeln zu können.

## Introduction

Intellectual disability (ID) is a substantial impairment of cognitive and adaptive function with an onset in early childhood. An objective measure is an intelligence quotient (IQ) < 70. Depending on the IQ measure, ID can be subdivided into mild (IQ: 55–70), moderate (IQ: 40–55), severe (IQ: 25–40) and profound (IQ < 25).

Intellectual disability often co-occurs with other mental conditions, e. g. depression, attention deficit/hyperactivity disorder (ADHD) and autism spectrum disorder (ASD). ID can be subdivided into non-syndromic and syndromic forms, the latter with additional clinical signs such as organ malformations. In many cases, the cause remains unknown owing to clinical and genetic heterogeneity.

The term “mental retardation” was used for many years to diagnose individuals with a reduced IQ. This term was considered to be no longer politically correct as “retard” stigmatizes those individuals [1]. Thus, the term ID is used in this review.

Non-genetic causes of intellectual disability include pre- and postnatal infections, perinatal asphyxia, maternal conditions such as diabetes or phenylketonuria, premature birth, environmental factors (e. g. iodine deficiency, malnutrition), metabolic causes and teratogens (e. g. intrauterine exposure to alcohol, drugs, environmental chemicals). However, most of the environmental factors depend on maternal lifestyle and healthcare.

This review mainly focuses on the genetic causes of autosomal dominant ID, which are responsible at least for the more severe forms of ID. Autosomal recessive and X‑chromosomal ID and chromosomal aberrations are not included in this review. For this topic please have a look at Rami Jamra’s article “Genetics of autosomal recessive intellectual disability”, Andreas Tzschach’s article “X-chromosomale Intelligenzminderung” and Hartmut Engels’ article “Strukturelle Chromosomenstörungen bei Intelligenzminderung” in this issue.

Intellectual disability is mostly sporadic in Western populations where consanguinity of the parents is rare. In these individuals *de novo* copy variations and *de novo* mutations in single genes detected by whole exome sequencing (WES) or whole genome sequencing (WGS) are causative. *De novo* mutations account for 42% of the architecture of severe ID and it is hypothesized that the mutations might be equally split between loss of function and altered function [2].

At the beginning of the next generation sequencing era, ID was distinguished in syndromic and non-syndromic ID. As many of the phenotypes caused by mutations in ID genes are extremely variable, non-syndromic ID can be found at the mild end and syndromic ID at the more severe end for mutations in the same gene, e. g. Coffin-Siris syndrome [3, 4]. Therefore, subdividing ID in these two groups is not warranted anymore, although associated clinical findings can help to find the causative gene.

*De novo *mutations occur predominantly in the sperm cell or egg and result in an embryo with a germline mutation, but post-zygotic point mutations in the index patient or low-level mosaicism in one parent have also been described [5].

As ID is one of the most common themes in genetic services, the following article provides an update on this topic.

## Prevalence of autosomal dominant ID

The prevalence of ID depends on the portion of consanguineous and non-consanguineous marriages. In Western populations with a low rate of consanguineous marriages, the prevalence of ID with an IQ < 70 is estimated to be 1.5–2%; for severe ID (IQ < 50) it is 0.3–0.5% [6]. A very recent review of 20 relevant articles gives a similar range of 0.05 to 1.55% for ID [7]. Two Australian surveys reported that the ID prevalence is age dependent with a prevalence of 1/303 in an age range of 20 to 50 years of the affected individuals, but increased to 1/70 if the age range was 6–15 years [1].

Developmental disorders caused by *de novo* mutations are reported to have an average prevalence of 1/448 (both parents aged 20) to 1/213 births (both parents aged 45) [2]. ID itself is not a rare disorder, but each subgroup – caused by mutations in different genes – is rare.

## Number of genes known to cause autosomal dominant ID

It is hypothesized that mutations in more than 1000 different genes might cause ID, some authors propose that the correct number is up to 2000 [1]. The estimates differ from paper to paper but more than 400 genes for autosomal dominant ID have been identified so far [8–10]. The SysID database (http://sysid.cmbi.umcn.nl/, [9]) lists 654 disease genes causing 535 diseases (as of 1 July, 2018), including 310 candidate genes. Fitzgerald et al. hypothesized in 2015 that most of the genes for autosomal dominant ID might have been identified [11].

In contrast, autosomal recessive ID (ARID) seems to be more heterogeneous and it is estimated that more than 3000 genes are involved [12]. It is interesting to note that 6% of individuals with ID born to first cousins or closer had a plausibly pathogenic *de novo* mutation [2]. For X‑linked ID, only 141 genes have been published up to 2017 [13].

## Frequently mutated genes in autosomal dominant ID

It is challenging to find reliable information on this topic. Most of the papers do not give estimates for the frequency of mutations within their identified genes.

According to the DDD study the most commonly mutated genes in their cohort are *ARID1B* (Coffin-Siris syndrome), *SCN2A, ANKRD11* (KBG syndrome), *SATB2* (Glass syndrome), *SYNGAP1, DYRK1A, MED13L, STXBP1, CTNNB1, KCNQ2, KMT2A* (Wiedemann-Steiner syndrome), *FOXP1, PACS1* (Schuurs-Hoeijmakers syndrome), *SMARCA2* (Nicolaides-Baraitser syndrome) and *WDR45* [11], all autosomal dominant genes besides WDR45. Some of these commonly mutated genes are causative for well-known syndromes and can now be identified in milder and more unspecified ID phenotypes.

Although the DDD consortium wrote in 2015 that the identification of genes for autosomal dominant ID is nearly completed, this seems not to be true. If all the publications in the *American Journal of Human Genetics and Nature Genetics* from January to June 2018 are analysed, 13 new genes for autosomal dominant ID have been published, namely *WASF1, TRAF7, TLK2, RORA, NAA15, PACS2, DPF2, CDC42, RHOBTB2, DHX30, FBXO11, BCL11B* and *BRD4*. It may be calculated that about 30 new genes for autosomal dominant ID will be identified by the end of this year, which is 10% of the known ID genes, excluding those that are only candidate genes (Table 1; [9]).

**Table 1:** Estimated frequencies for mutations in commonly mutated genes in intellectual disability (ID) cohorts

Gene	Frequency (%)
*ARID1B*	0.5–1 [4, 11]
*SCN2A*	0.5–16 [11, 14, 15]
*ANKRD11*	0.5–1 [11]
*SATB2*	0.3 [11]
*SYNGAP1*	0.5–16 [11, 15, 16]
*DYRK1A*	0.44 [17]
*MED13L*	0.5–1 [11]
*STXBP1*	0.5–16 [11, 15]
*CTNNB1*	1–2 [18, 19]
*KCNQ2*	3 [20]

## Diagnostic rates in autosomal dominant ID

These data vary from 20 to 60% according to several factors, e. g. detailed clinical characterization of patients, heterogeneity of the condition, the applied technology, including coverage and the bioinformatic workflow and analyses of clinical data. A very recent paper from the DDD study summarized that trio WES has a diagnostic yield of 50% for affected individuals [21].

The technology used differs in research and diagnostics and can comprise panel sequencing, WES, WGS and also the third generation long-read sequencing with the PacBio and Nanopore technologies and Bionano mapping technologies [22], which have not been used for diagnostic purposes so far. Proof of principles for these new technologies have been published and could demonstrate that additional mutations were detected with these methods, although extended experience for human data is still lacking. However, it may be expected that the mutation detection rate for ID will increase further (Table 2).

**Table 2:** Mutation detection rate for ID in selected studies

Reference	Vissers Lelm et al., 2010 [23]	De Ligt et al., 2012 [18]	Rauch et al., 2012 [15]	DDD study, 2015 [11]	DDD study et al., 2017 [2]	Wright et al., 2018 [21]
Number of patients analysed (*n*)	10	100	51	1133	148	1133
Method applied	Trio WES	Trio WES	Trio WES	Trio WES	Trio WES	Trio WES
Detection rate (%)	60	16	45–55	27	31	40–43

*WES* whole exome sequencing

Some of the early papers applying trio WES for the identification of mutations in ID included individuals with severe ID and thus the mutation detection rate was high, although the bioinformatic pipeline was in the fledgling stages. The more recent papers had larger and more heterogeneous cohorts of patients, especially the DDD study, and the mutation detection rate also increased owing to the optimization of the pipelines. This could be demonstrated by re-analysing the previous DDD cohort [21]. However, the mutation detection rate depends on inclusion criteria, the quality of clinical and sequencing data and on the bioinformatic pipelines.

## Increase in the mutation detection rate after re‑analysing the data

A few papers have been published dealing with re-analyses of WES data. Costain et al. [24] re-analysed WES data of 100 patients 2 years later. They identified seven more causative variants in 64 previously undiagnosed patients and increased the diagnostic yield to 41%. Wright et al. [21] re-analysed the data of 1133 children after 4 years and were able to diagnose an additional 182/1133 patients. The diagnostic yield increased from 27 to 40%. Alfares et al. [25] reported in a study of 108 patients that WGS achieved an only 7% higher detection rate than WES. They described that in 4 patients, the variants were missed for different reasons in the first-run analyses of WES data.

One can conclude that the re-analysis of WES data is recommended before performing WGS.

## Disease-causing mutations in more than one gene

It is well-known that in some individuals, the ID is caused by mutations in more than one gene. Fitzgerald et al. reported 17/148 (11%) individuals showing a composite phenotype due to disease-causing mutations in two genes [2]. In a retrospective analysis of 7374 consecutive patients, not only ID patients, a molecular diagnosis was established after whole exome sequencing in 2076 patients and 4.9% of them had diagnoses that involved two or more disease loci [26].

## Relevance of gender in autosomal dominant ID

Male patients with ID have a statistically significantly lower risk of carrying a pathogenic *de novo* mutation compared with female patients [2, 11], which was also reported for autism [27]. This can be explained by the fraction of mutations in X‑linked genes explaining the ID in these male individuals. For X‑linked ID in male patients, please refer to the article by Andreas Tzschach “X-chromosomale Intelligenzminderung” in this issue and for X‑linked ID in female patients the article by Christiane Zweier “X-gebundene Entwicklungsstörungen im weiblichen Geschlecht”.

## Relevance of mosaicism in autosomal dominant ID

As Sanger sequencing does not provide reliable information for distinguishing between somatic and germline mutations, little is known about the proportion of *de novo* mutations that occurred during gametogenesis and the postzygotic mutations. Acuna-Hidalgo et al. analysed 107 *de novo* mutations in 50 parent-child trios concerning this question. They observed with WGS that 7/107 (6.5%) mutations were mosaic mutations in the blood, which were previously suspected to be germline mutations. Krupp et al. identified mosaic *de novo* mutations in 4.2% of individuals with autism spectrum disorder [28]. This is of great importance for individuals as low-level mosaicism (1% in affected tissues) can be clinically significant, e. g. Sturge-Weber syndrome.

In addition, 4/4081 variants, which were classified to be *de novo*, were also present in the blood of one parent. Thus, parental mosaicism was the cause of these variants [5].

These findings have an impact on the genetic counselling of these families. If the mutation occurs post-zygotically in the child, the recurrence risk for further children of the parents is lower than expected for germline mutations. In contrast, if one parent shows low-level mosaicism, the recurrence risk for further children is higher than in *de novo* germline mutations.

Acuna-Hildalgo et al. concluded that there is a need for higher coverage in WES/WGS to detect mosaicism correctly [5]. One should also keep in mind that in most of the studies only DNA from blood was analysed. More studies on other tissue samples, e. g. fibroblasts, saliva, urine, will give further insights into the relevance of mosaicism in ID. Especially for ID, the tissue of choice – brain tissue – cannot be analysed to find low-grade mosaicism that is causative for ID.

## Relevance of parental age in autosomal dominant ID

It is well-known that advanced maternal age leads to an increase in chromosomal aberrations and an advanced paternal age to an increase in *de novo* point mutations. Only some of the papers on ID evaluated parental age of individuals with ID. Paternal age was only weakly associated with a risk of the child having a *de novo* mutation, but focusing on the minority of *de novo* mutations that were truncating and missense variants in known ID genes limited this effect. However, analysis of all high-confidence exonic and intronic autosomal *de novo* mutations (*n* = 8409) revealed a strong effect of paternal age [2] and a milder effect of maternal age [2, 29]. There seems to be a paternal effect of 0.03606 *de novo* mutations per year and a maternal effect of 0.0172 *de novo* mutations per year [2].

## Diagnostic approaches

Trio WES is the most efficient and convenient diagnostic tool for sporadic individuals with ID in general and for identifying autosomal dominant/*de novo* mutations specifically. In the near future, the bioinformatic pipelines will be optimized for WGS and it can be hypothesised that this technique might then become the method of choice. It is only a matter of time before we use long-read sequencing and long-read mapping to resolve those individuals without mutations in WES and short-read WGS, as has been demonstrated for single individuals [30].

## Outlook

As discussed above the mutation detection rate is limited to 20–60%, mostly within the coding region of the exome. In principle, mutations can be detected in the non-coding regions of the genome when applying WGS. In practice, we are not able to detect all disease-causing variants in intronic regions, like enhancers, repressors or insulator mutations in intergenic regions so far. Novel algorithms were developed and must be developed in the future to identify not only intronic variants, but also exonic variants affecting splicing or generating novel splice sites [31, 32]. Epigenetic changes [33], di-/polygenic inheritance and parental germline mutations should also be taken into consideration when trying to resolve ID in general.

In addition, it may be speculated that the 350 as yet unidentified genes for autosomal dominant ID might cause rare diseases and that identification might benefit from large national and international collaborations.
